# Overcoming viral escape with vaccines that generate and display antigen diversity in vivo

**DOI:** 10.1186/1743-422X-4-125

**Published:** 2007-11-22

**Authors:** Albert García-Quintanilla

**Affiliations:** 1 Atanasio Barrón 14, P4-5A, 41003 Sevilla, Spain

## Abstract

**Background:**

Viral diversity is a key problem for the design of effective and universal vaccines. Virtually, a vaccine candidate including most of the diversity for a given epitope would force the virus to create escape mutants above the viability threshold or with a high fitness cost.

**Presentation of the hypothesis:**

Therefore, I hypothesize that priming the immune system with polyvalent vaccines where each single vehicle generates and displays multiple antigen variants *in vivo*, will elicit a broad and long-lasting immune response able to avoid viral escape.

**Testing the hypothesis:**

To this purpose, I propose the use of yeasts that carry virus-like particles designed to pack the antigen-coding RNA inside and replicate it via RNA-dependent RNA polymerase. This would produce diversity *in vivo *limited to the target of interest and without killing the vaccine vehicle.

**Implications of the hypothesis:**

This approach is in contrast with peptide cocktails synthesized *in vitro *and polyvalent strategies where every cell or vector displays a single or definite number of mutants; but similarly to all them, it should be able to overcome original antigenic sin, avoid major histocompatibility complex restriction, and elicit broad cross-reactive immune responses. Here I discuss additional advantages such as minimal global antagonism or those derived from using a yeast vehicle, and potential drawbacks like autoimmunity. Diversity generated by this method could be monitored both genotypically and phenotypically, and therefore selected or discarded before use if needed.

## Background

Viral reverse transcriptases and RNA-dependent RNA polymerases (RDRP) show the highest mutation rates found in nature. Diversity generated allows these viruses to evade host defenses and poses a key problem in vaccine design. Infected individuals develop a titanic fight to avoid viral escape. However, above certain diversity threshold the immune system is unable to control the virus and collapses [1,2]. Likewise, inverse relationship between vaccine efficacy and virus divergence has been demonstrated [3] and may explain why no vaccine exists against the highly variable HIV or hepatitis C virus, in contrast to other viruses such as influenza virus (where formulations need to be updated yearly to include predominant circulating strains) or hepatitis B virus (where immunologically targeted regions remain conserved due to the viral fitness cost and the vaccine is feasible and broadly effective).

Current vaccine strategies to overcome diversity include the use of (I) conserved or consensus epitopes [4,5], (II) chimeric antigens containing fragments from diverse populations [6-8], and (III) the inclusion of multiple strain variants of the same antigen [9]. Here I propose a new kind of vaccines that generate diversity *in vivo*.

## Presentation of the hypothesis

I hypothesize that priming the immune system with polyvalent vaccine candidates where each single vehicle generates and displays multiple antigen variants *in vivo*, is safe and will elicit a broad and long-lasting immune response able to avoid viral escape. This strategy is different from peptide cocktails synthesized *in vitro *and polyvalent strategies where every cell or vector displays a single or definite number of mutants.

## Testing the hypothesis

In order to generate such diversity *in vivo *I propose the use of recombinant yeasts that carry virus-like particles (VLPs) designed to pack the antigen-coding RNA inside and replicate it via RDRP. The VLPs can be coded *in cis *and pack their own RNA with or without heterologous sequences inserted, or be supplied *in trans *by a vector and pack a heterologous RNA that bears the appropriate *cis *packing and replication motifs. A particular example of this last would be the use of *S. cerevisiae *carrying L-A totivirus VLPs that pack and replicate the RNA coding for an HIV epitope.

Diversity will take place only within the RNA of interest every time the RDRP replicates it, while keeping the genome unchanged. Degree of variation will be a function of the RDRP mutation rate, number of cycles (time), target length and average of VLPs per cell. For a conservative replication mechanism, every daughter cell will accumulate most of its diversity history. These mutations will occur randomly whenever not toxic or essential for the cell or VLPs, and will be kept under biological parameters. However, only sequences that share enough homology to be packed and replicated will be transmitted. The yeast cell wall will avoid cellular lysis, and VLPs will spread by cytoduction, without killing the yeast. The target RNAs will be translated by the cellular machinery and their products displayed on the cellular surface [10], secreted outside or kept inside the cells. Multiple yeasts may be designed using consensus, conserved, variable or mosaic target RNA sequences as starting points.

## Implications of the hypothesis

This strategy is supported by a mathematical model [11] that predicts that a vaccine will fail if it does not protect against a sufficiently large fraction of HIV strains, no matter how immunogenic it is. In agreement with this, other teams have proposed to include most of the diversity for a given epitope based on peptide combinations synthesized *in vitro *[12-14]. However, these candidates require adjuvants to elicit an immune response and their synthesis is complex. Similarly, polymorphic proteins or DNA vaccines generated *in vivo *were suggested before [15] but up to date nobody had solved how to create diversity without killing the host neither mutating its genome. Thus, infection of cell cultures or animals used for attenuated or inactivated vaccines generate limited variation and kill the host, while *in vivo *evolution strains like *E. coli *XL-1 Red (Stratagene) or protocols with mutagens and retrotransposons mutate the host genome.

Polyvalent vaccines containing multiple variants of an epitope (I) elicit broader cross-reactive immune responses than candidates with fewer variants [9,16], (II) overcome original antigenic sin that may result from sequential exposure to antigen variants [16], (III) avoid lack of cellular response to certain epitopes due to major histocompatibility complex (MHC) restriction [17] and, (IV) may suppress T-cell antagonism when peptides are related [18]. Similar results would be expected also for antigenic diversity generated *in vivo*.

Some authors have suggested that simultaneous administration of antigen variants could be subject to T-cell antagonism [19]. However, only some amino acid substitutions are antagonist and occur mainly within the positions that bind the T-cell receptor, while the others are neutral or agonist [20]. These authors [19] also indicate that the stronger the response to a particular epitope, the smaller the relative effect of antagonism. Therefore, this interference should be less in immunodominant epitopes. A polyvalent vaccine that broadens the immune response against all epitope variants will minimize global antagonism and will avoid viral escape, even if some altered peptide ligands reduce immunodominance of the original epitope by lowering the peptide-MHC complex stability.

Another potential concern of this approach is that T cells require around 100–200 identical MHC-peptide complexes on the target cell to be activated. This implies that each new variant needs to reach a minimum number or percentage to raise a response against it. This should not be a problem since every RNA is translated several times. Also, once a mutation arises, the probability of a new change within the radius of MHC-displayed peptides containing such mutation is very low, thus ensuring enough copies of the variant occurred. Hence, even if two RNAs with mutations alongside are different they may share several identical T-cell epitopes.

Similarly, each particular mutant could be too rare to stimulate a broad humoral immunity. The first interaction between a B cell and a pathogen is usually multivalent, with many weak interactions between multiple B-cell receptors and pathogen proteins "adding up". But if a particular B cell can only bind (weakly) to a few copies of antigen on a yeast cell, that B cell might never become activated. However, this situation may end up being good because only the B cells that can recognize many antigen mutants would expand clonally. This potential advantage may be lost in current polyvalent vaccines where "enough" amount of every variant is displayed, thus favouring broad antibody responses instead of broadly neutralizing antibodies.

There is a common thinking that vaccines containing high number of variants would include also antigens that show molecular mimicry to self-antigens and therefore could cause autoimmunity. Despite examples of autoimmune diseases caused by infections exist, recent studies show that molecular mimicry has been overvalued and that by itself is not enough to break immune tolerance, illustrating that these events are dose-dependent and require other mechanisms, like bystander activation and antigenic persistence [21,22], or a previous underlying process in susceptible individuals [23]. Under normal conditions the immune system prevents the formation of self-reactive antibodies, and when they arise they are usually transient. In fact, some of the broadly neutralizing antibodies against HIV are polyspecific antibodies that react with self-tissues [24]. Paradoxically, two drugs (Copaxone and Peptimmune-2301) based on the use of random sequence peptide mixtures reduce the frequency of autoimmune multiple sclerosis relapses. In the worst possible scenario, susceptible individuals could be tested individually before applying or discarding vaccination. If the risk to trigger autoimmunity becomes unacceptable for prophylactic use, it still would be a therapeutic alternative for diseases without a cure. This is the case for cancer treatments under clinical trials using antibodies against self-antigens [25].

The final drawback is relative to current manufacturing and quality regulations that require every lot to be fully characterized and reproducible in order to be marketable. This is intrinsically difficult for random diversity, but new emerging technologies like microarrays are now available and let genotype characterization of thousands of sequences in a single experiment, while other well-known protocols, such as FACS, allow phenotype selection or disposal of specific proteins and cells, thus guarantying the maximum presence of desired variants, and the exclusion of unwanted ones that could bind to autoimmune serum. In the best setting, an extraordinary product with exceptional results may force current policies to change in order to outweigh all other considerations. In the opposite situation, it would serve as proof of concept.

Finally, the yeast vehicles allow post-translational processes that bacterial vectors cannot, economical production, multiple doses, and dried formulations to omit the cold chain. They also exert a strong adjuvant effect [26] and can be administered orally to target the gut-associated lymphoid tissue [27] (which comprises over 70% of the immune cells and represents the main reservoir and replication site for HIV and other viruses). In addition, the system proposed includes the use of VLPs, which provide an efficient strategy to raise immune responses toward desired epitopes [28].

## Competing interests

AGQ has applied for a patent covering the methods described here to generate and display antigenic diversity *in vivo*.
